# Integrating ethics into infectious disease graduate training: a multidimensional framework for public health practice

**DOI:** 10.3389/fpubh.2026.1744330

**Published:** 2026-02-18

**Authors:** Xiaohao Wang, Hu Li, Tiantian Li, Shan Zhong

**Affiliations:** Department of Infectious Diseases, Key Laboratory of Molecular Biology for Infectious Diseases (Ministry of Education), Institute for Viral Hepatitis, The Second Affiliated Hospital, Chongqing Medical University, Chongqing, China

**Keywords:** curriculum innovation, digital health ethics, ethical leadership, genomics ethics, graduate medical education, infectious disease ethics (IDE), multidimensional ethical framework, public health practice

## Abstract

Through a narrative review and synthesis of the global status of Infectious Disease Ethics (IDE) education, this paper proposes positioning IDE as a core competency in graduate training and constructs a three-dimensional integrated model of “Theory-Practice-Assessment.” Drawing on the experience of the OPENING project by the European Society of Clinical Microbiology and Infectious Diseases (ESCMID), it emphasizes that the ethical framework must adapt to the paradigm shifts brought about by emerging technologies such as genomics. This model not only addresses the gaps in IDE education exposed by COVID-19 but also provides solutions to ethical challenges in fields like digital health and precision medicine, offering a practical pathway for the reform of global infectious disease graduate education.

## 1 The urgency of infectious disease ethics education

### 1.1 Ethical vacuum and lessons from COVID-19

The COVID-19 pandemic has brought ethical dilemmas in the field of infectious diseases to the forefront, directly exposing systemic deficiencies in existing IDE education. A global survey shows that only 32% of infectious disease (ID) professionals have received systematic ethical training, with significant variations in training quality (1–3). The disparity across regions is particularly striking: the training coverage rate reaches 58% in developed European countries and 45% in North America, while it is merely 11 and 9% in low-income countries in Africa and Southeast Asia, respectively. This imbalance has exacerbated inequalities in global public health responses (1).

The lack of structured guidance for resolving ethical conflicts between bedside decisions and public health interventions has led to numerous controversies in practice (4). During the peak of the pandemic, many countries faced shortages of medical resources such as ventilators and intensive care beds. There were neither unified ethical standards nor systematic decision-making training for professionals when allocating resources between older adults and young patients, or between critically ill and moderately ill patients (4). Meanwhile, issues such as balancing individual freedom with public health in quarantine policies and disputes over fairness in vaccine distribution have all highlighted the practical necessity of IDE education (4, 5).

### 1.2 New challenges arising from technological innovation

The widespread application of genomics technology in infectious disease surveillance has challenged the traditional definition of patient privacy (3). Taking molecular epidemiological research on tuberculosis as an example, tracking the source of infection through gene sequencing involves collecting a large amount of personal and family genetic information of patients. The scope of data sharing, storage duration, and boundaries for secondary use of such data lack clear ethical norms, which may lead to genetic discrimination or privacy breaches (3).

The popularization of digital health tools has also created new informed consent dilemmas (3, 5). Contact tracing apps played a crucial role in pandemic prevention and control, yet some tools failed to fully inform users about the scope and purpose of data collection. Users often authorized platforms to access sensitive information such as location and communication records without full awareness. This “passive informed consent” violates the fundamental principles of ethics, undermining public trust and laying hidden risks for data abuse (5).

### 1.3 The unique position of graduate education

Unlike undergraduate education, which focuses on imparting ethical knowledge, graduate medical education (including residency, fellowship, and advanced degree programs in infectious diseases) should prioritize the cultivation of “ethical leadership” (6, 7). Undergraduate programs typically cover basic content such as the four core principles of medical ethics, while graduate students—who will be the backbone of future infectious disease prevention and control—need the ability to lead ethical decision-making and coordinate multi-stakeholder interests in complex scenarios. For instance, in cross-regional epidemic responses, they should be able to balance resource needs across different regions and develop fair and feasible intervention plans (7).

The interdisciplinary nature of infectious disease ethics dictates that graduate education must integrate clinical microbiology, public health policy, and philosophical ethics (8, 9). Infectious disease prevention and control not only involves medical-technical aspects of diagnosis and treatment but also requires consideration of the legitimacy of policy formulation, the fairness of social impacts, and the acceptability of public perception (9). Therefore, graduate training must break down disciplinary barriers and foster comprehensive literacy for analyzing and solving problems from medical, policy, and ethical perspectives.

### 1.4 Methodology of this review

This paper adopts a narrative review approach to synthesize existing literature, policy documents, and educational frameworks related to infectious disease ethics education. Literature was identified through PubMed, Web of Science, and Google Scholar using keywords including “infectious disease ethics,” “ethics education,” “graduate medical education,” and “public health ethics.” Sources were selected based on relevance to curriculum design, ethical frameworks, and international educational practices. The review aims to provide a comprehensive overview and propose a structured framework rather than a systematic meta-analysis.

## 2 Limitations and innovations of existing educational models

### 2.1 Shortcomings of traditional approaches

Traditional IDE education is plagued by fragmentation, with ethical content often confined to a single “professional ethics” module, lacking systematicity and relevance (10, 11). Most medical schools only offer 2–4 h of ethical lectures in graduate curricula, covering a narrow range of topics that are disconnected from core aspects of infectious disease clinical practice and public health interventions (10). As a result, students struggle to apply ethical knowledge to practical work.

The absence of an assessment system further hinders the improvement of educational quality. The Accreditation Council for Graduate Medical Education (ACGME) has established clear ethical competency standards for specialties like hematology, but no such unified framework exists for IDE (12). Existing assessments primarily rely on theoretical exams, focusing on memorization of ethical principles rather than evaluating practical decision-making skills or the ability to resolve ethical conflicts, failing to objectively reflect students’ ethical literacy (12).

### 2.2 The role of emerging educational technologies

Large language models such as ChatGPT present a double-edged sword in ethical case discussions (13). These tools can quickly generate diverse ethical dilemma scenarios and simulate the perspectives of different stakeholders, providing students with rich discussion materials—especially valuable for institutions with limited resources. However, AI lacks the human-specific capacity for value judgment and empathy; it cannot guide students to deeply reflect on the humanistic concerns behind ethical decisions or cultivate their moral intuition (13). Thus, it cannot replace the core role of human mentors.

The development and application of online educational resources have opened new avenues for IDE education. A genomics training module designed for members of ethics committees covers core content such as ethics of genetic data and norms for informed consent through video lectures and interactive case analysis. In pilot applications across more than 20 countries, the accuracy rate of participants’ ethical decision-making increased by 40%, demonstrating the feasibility of the online model (14). Additionally, virtual reality (VR) technology can simulate ethical scenarios in emergent epidemics, allowing students to practice decision-making in immersive environments and serving as an effective supplement to traditional teaching (1).

### 2.3 Comparison of international practices

Europe has operationalized ethical principles through ESCMID’s OPENING project (Operationalizing Ethics in Infectious Diseases), which integrates ethics into Clinical Practice Guidelines (CPGs), providing concrete tools for IDE education (2). The organization has developed a fairness assessment form encompassing 8 core indicators, including resource allocation, service accessibility, and risk–benefit ratio. Students can practice using this form with real cases, translating abstract fairness principles into actionable decision-making steps (2). This “principle-tool-practice” model effectively enhances the practicality of ethical education.

The American model focuses on integrating community ethical practice, though it should be noted that practices vary across institutions and regions. Teaching Health Centers place residents at the forefront of community epidemic prevention and control, allowing students to confront ethical challenges in real-world work (15). For example, during community vaccine administration, students must address issues such as vaccine allocation during shortages, answer public questions about vaccine safety, and coordinate vaccination priorities across age groups (15). Through reflective practice, they deepen their understanding of ethical principles and develop communication, coordination, and decision-making skills.

In addition to European and American models, Asian contexts—particularly China—also offer valuable approaches. China’s dual-tutor system, which pairs ethics scholars with clinical mentors, provides a structured mechanism for integrating ethics into clinical training (16). This model emphasizes localized adaptation of ethical principles to regional public health challenges, such as large-scale outbreak response and resource allocation in densely populated settings.

While the European model emphasizes the application of standardized tools and the American model prioritizes practical experience, these approaches, along with regionally adapted models like China’s, collectively embody the core idea of “integrating theory with practice,” offering valuable insights for global IDE education reform.

## 3 Construction of a three-dimensional integrated framework

The proposed framework comprises three interconnected dimensions: Theoretical, Practical, and Assessment. This structure ensures that ethical training is not only knowledge-based but also skill-oriented and evaluable, thereby fostering measurable competency development in trainees.

### 3.1 Theoretical dimension

IDE theoretical teaching should expand beyond the traditional “four core principles” (autonomy, non-maleficence, beneficence, and justice) to include seven categories: genomic justice, digital ethics, global health justice, community engagement ethics, research ethics, policy ethics, and cultural sensitivity ethics (3, 10). This expansion is justified by the evolving landscape of infectious disease challenges, where issues like data privacy, global equity, and community trust are as critical as bedside ethics (3, 8). The seven categories were selected to cover the spectrum from individual patient care to global health governance. It is recommended that 2–3 teaching hours be allocated to each category, with a total of no fewer than 14 h to ensure comprehensive theoretical coverage. This allocation is based on curriculum design principles for competency-based graduate education, ensuring sufficient depth for each topic while remaining feasible within standard program structures (10). This proposed structure of seven thematic areas, while derived from a synthesis of current ethical challenges in the literature (3, 8, 10), represents a curricular proposal based on our expert analysis and has not yet undergone formal validation through methods such as a Delphi consensus process. It is intended as a comprehensive framework to guide curriculum development.

Genomic justice focuses on the fair use of genetic data and accessibility of genetic technologies; digital ethics addresses issues such as health data privacy protection and fairness in algorithmic decision-making; and global health justice emphasizes the right to resource allocation and technology access for low-income countries in global infectious disease prevention and control (3). Through systematic teaching, students will develop a multidimensional ethical thinking framework.

Local adaptation is a key component of theoretical teaching. China’s dual-tutor system provides a solid foundation for collaboration between ethics scholars and ID experts (16). A “ethics tutor + clinical tutor” joint guidance model can be adopted: ethics tutors are responsible for theoretical teaching and guidance on ethical analysis methods, while clinical tutors, drawing on cases from infectious disease diagnosis/treatment and public health practice, explain the application scenarios of ethical principles—achieving deep integration of theory and practice. For example, when discussing the ethics of tuberculosis genetic surveillance, clinical tutors can share practical cases of privacy protection, while ethics tutors analyze the ethical conflicts in these cases and their resolution pathways from a theoretical perspective.

### 3.2 Practical dimension

Practical teaching should follow a structured “basic level-advanced level” pathway to gradually enhance students’ practical ethical competence. The rationale for this progression is to scaffold learning from skill-based ethical awareness to complex ethical decision-making in simulated and real-world settings.

The basic level requires embedding ethical considerations into technical training. For example, in point-of-care ultrasound (POCUS) training, gender sensitivity education should be incorporated—emphasizing privacy protection and cultural respect in ultrasound examinations for female patients (17). In pathogen detection technology training, ethical norms for communicating test results should be covered, including how to explain results to patients, protect patient privacy, and respond to patients’ psychological reactions (17).

The advanced level focuses on simulation training and on-site practice, with an emphasis on cultivating ethical decision-making skills in emergent epidemics. Scenario simulations can be designed to replicate situations such as ventilator shortages during the COVID-19 pandemic or the formulation of quarantine policies following the outbreak of an unknown infectious disease. Students are divided into groups to play roles such as clinicians, public health officials, and patient representatives, develop resource allocation plans or intervention policies, and conduct ethical justifications (18). Additionally, students can be arranged to participate in on-site tasks such as ethics committee work and community infectious disease prevention, allowing them to address ethical conflicts in real-world settings.

### 3.3 Assessment dimension

The third dimension of the framework focuses on evaluating ethical competency through structured tools and processes.

To systematically evaluate ethical competency, we propose a preliminary 10-item core assessment framework for IDE. The design of this framework is inspired by the use of structured checklists and rubrics in competency-based medical education, which are proven tools for making complex skills like ethical reasoning observable and assessable (19, 20). Furthermore, its systematic approach is analogous to ethical review frameworks employed in fields such as forensic epidemiology, where structured guidelines help navigate shared core concerns—including privacy, justice, and accountability during public health investigations (21, 22). Its structured checklist approach provides a validated model for translating ethical principles into observable, assessable behaviors. The proposed tool includes adequacy of informed consent, effectiveness of privacy protection, fairness of resource allocation, rationality of risk–benefit ratio, demonstration of cultural sensitivity, avoidance of conflicts of interest, degree of community engagement, legitimacy of policy formulation, transparency of communication, and traceability of decisions. The selection of items was refined by the authors’ collective expertise in infectious diseases and bioethics. Its future application and refinement should involve rigorous validation through methods such as Delphi surveys with international IDE experts or pilot testing in training programs (21, 23).

The proposed 10-item assessment tool is designed to operationalize and evaluate the core competencies outlined in the seven theoretical categories. For instance, ‘fairness of resource allocation’ and ‘global health justice’ assess competencies in Global Health Justice; ‘adequacy of informed consent’ and ‘transparency of communication’ relate to Digital Ethics and Research Ethics; ‘demonstration of cultural sensitivity’ aligns with Cultural Sensitivity Ethics; and ‘legitimacy of policy formulation’ and ‘degree of community engagement’ correspond to Policy Ethics and Community Engagement Ethics. This ensures that assessment is directly aligned with the taught curriculum.

### 3.4 Institutional guarantee

Curriculum accreditation is critical to ensuring educational quality; it is recommended that the World Health Organization (WHO) develop minimum standards for IDE graduate education (12). These standards should specify key elements such as core curriculum content, the ratio of theoretical to practical hours, requirements for implementing the dual-tutor system, and assessment methods—providing a unified reference for IDE education worldwide. Meanwhile, regional IDE education accreditation bodies should be established to regularly evaluate the training quality of institutions and promote the implementation of educational standards.

Furthermore, integrating research ethics training, particularly concerning implementation science, into the core curriculum is essential. Implementation science focuses on how to effectively apply infectious disease prevention technologies and policies in real-world settings, with associated ethical issues including: fairness in promoting new technologies in resource-constrained regions, community acceptance of intervention measures, and coordination of stakeholders in policy implementation (5, 13). Targeted training will enable students to identify and address ethical risks in the implementation process.

Establishing a faculty development system is a key pillar of institutional guarantee. An international collaborative network can be used to provide training for IDE educators worldwide, covering content such as ethical teaching methods, case development, and the use of assessment tools. Additionally, ethics scholars and ID experts should be encouraged to collaborate on educational research and jointly develop localized teaching resources and case banks to enhance the relevance and practicality of education.

## 4 Conclusion

Infectious disease ethics education needs to transform from an “add-on” to an “infrastructure,” becoming a core component of infectious disease graduate training. The three-dimensional integrated “Theory-Practice-Assessment” framework constructed in this paper provides a systematic solution for cultivating the next generation of ID experts with ethical leadership, through comprehensive theoretical coverage, structured practical training, and sound institutional guarantees.

This framework incorporates international experiences from regions such as Europe and North America while considering local adaptation needs. It can address ethical challenges brought about by emerging technologies like genomics and digital health, and respond to the educational gaps exposed by COVID-19. By establishing a global collaborative network (drawing on the ESCMID case 2) and leveraging innovative educational technologies, the framework can be implemented worldwide.

However, IDE education reform still faces financial and policy obstacles. Low-income countries generally lack faculty and funding for ethical education, requiring support from international organizations and high-income countries. In some regions, IDE education is not sufficiently prioritized, and it has not been incorporated into mandatory graduate training requirements at the policy level (1, 12). In the future, global collaboration is needed to mobilize resources and strengthen policy advocacy to promote the popularization and quality improvement of IDE education. Ultimately, this will cultivate professionals capable of addressing full-chain ethical challenges “from pathogen to policy,” providing ethical safeguards for global infectious disease prevention and control.

## Data Availability

The original contributions presented in the study are included in the article/supplementary material, further inquiries can be directed to the corresponding author/s.
